# Editorial: Winning by design: technology-integrated learning, performance, and recovery in team sports

**DOI:** 10.3389/fpsyg.2026.2030251

**Published:** 2026-10-28

**Authors:** Bruno Figueira, José Luis Felipe, Rūtenis Paulauskas, Bruno Gonçalves

**Affiliations:** 1 Universidade de Évora, Escola de Saúde e Desenvolvimento Humano, Departamento de Desporto e Saúde, Évora, Portugal; 2 Universidade de Évora, Comprehensive Health Research Centre (CHRC), Évora, Portugal; 3 IGOID Research Group, Department of Physical Activity and Sport Sciences, University of Castilla-La Mancha, Toledo, Spain; 4 Performance Analysis Department, UD Las Palmas, Las Palmas de Gran Canaria, Spain; 5 Educational Research Institute, Education Academy, Vytautas Magnus University, Kaunas, Lithuania

**Keywords:** athlete monitoring, augmented feedback, perceptual-cognitive skills, recovery, representative learning design, team sports, technology acceptance, technology-integrated learning

Team sports require athletes to coordinate skilled actions and decisions under interacting tactical, physical, and psychological demands. Wearables, computer vision, artificial intelligence, and immersive simulation can enrich the information available to coaches, but practical benefit depends on valid interpretation. The Research Topic “*Winning by Design: Technology-Integrated Learning, Performance, and Recovery in Team Sports*” was conceived to connect these technologies with learning, performance, and recovery. Its scope emphasized validated key performance indicators, individualized workload and readiness assessment, and psychological mechanisms including attention, motivation, decision-making, and self-regulation. Representative learning environments and augmented feedback were considered alongside sleep, fatigue management, and the integration of physiological, biomechanical, tactical, and psychological information. The overarching objectives were to bridge laboratory and field settings, evaluate measurement quality and technological adoption, and support reproducible, transparent, and equitable implementation.

The Research Topic comprises nine articles, including eight original research papers and one opinion article, contributed by 34 authors affiliated with institutions in China, Germany, Israel, Italy, Japan, Lithuania, Portugal, and Spain. Published in Frontiers in Psychology and Frontiers in Virtual Reality, these contributions connect three domains: technology-integrated learning and training design; perception, action, and cognitive demands; and maturation monitoring and technology acceptance. Recovery and responsible implementation provide cross-cutting priorities.

## Evidence synthesis

Augmented information can support learning when it clarifies relevant actions. In a randomized youth-football study, He and Wei reported greater improvements in tested skills, intrinsic motivation, and follow-up retention with augmented-reality feedback than with verbal instruction. Competitive transfer remains to be established. Saiki et al. developed a mixed-reality basketball environment to practice collective coordination; novices improved positioning and timing. Nonsignificant expert differences between simulated and real performances do not demonstrate equivalence. Consistent with Chan et al. (2024) review, these findings justify testing simulation against live opposition rather than assuming match benefits.

Practice variability provides a complementary route to skill development. Burkaitė et al. compared differential and traditional shooting training in youth basketball. Benefits for selected shooting outcomes, without a between-group advantage in one-on-one scoring at retention, show that technical improvement and competitive transfer can diverge. Future work should identify variability doses that support adaptable shooting under opposition.

Perceptual demands help explain why transfer requires representative tasks. Using wearable eye tracking, Zhou et al. characterized task-dependent visual search in group rhythmic gymnastics; these descriptive findings support testing task-specific feedback, rather than prescribing a universal gaze strategy. Fan et al. found slower inhibitory oculomotor responses and altered distractor processing after prior self-control exertion. Whether these laboratory effects influence competitive decisions remains unresolved. Shalom et al. propose basketball cognitive–motor dual tasking in an opinion article, supplying a rationale for controlled intervention studies linking perceptual demands to skilled action.

Monitoring must accommodate development and engagement. Shang et al. describe Maturo, a smartphone prototype integrating growth and maturation monitoring. Preliminary feasibility and measurement associations warrant independent longitudinal validation across developmental contexts before selection or load decisions rely on its estimates. Wu et al. observed heterogeneous associations between tracker use and fitness improvement in adolescent footballers, moderated by acceptance and initial fitness. Wu and Zhang linked technology acceptance and virtual–real integration with adolescent participation through survey-based modeling. Together, these observational studies motivate personalized adoption strategies and prospective comparisons, without establishing causal performance benefits.

Direct recovery interventions remain underrepresented. Wearable data collection in injured adolescents is feasible, although clinical efficacy remains unresolved (Ransom et al., 2025). Cunha et al. (2023) identify sleep extension and naps as promising performance interventions, with limitations in study quality. Hülsdünker et al. (2025) found improved reactive agility after active motor-cognitive recovery vs. seated recovery without corresponding differences in measured physiological responses. Recovery research therefore needs subsequent performance outcomes alongside physiological monitoring.

An important observation emerging from this Research Topic is that technological integration has advanced more rapidly in learning and monitoring than in recovery optimization, highlighting a critical opportunity for future interdisciplinary research.

## Practical applications

The feedback and simulation studies support starting with a defined learning objective. Coaches can pilot augmented cues and variable practice, then assess retention without cues and transfer against live opponents, following the questions raised by He and Wei, Saiki et al., and Burkaitė et al. The developmental and acceptance findings of Shang et al. and Wu et al. likewise motivate combining validated measurements with maturation context and athlete experience. Individual baselines, perceived exertion, and sleep reports should inform explicit adjustment criteria; wearable outputs should prompt dialogue rather than automatic prescription. Across the studies included in this Research Topic, technology appears most valuable when supporting, rather than replacing, practitioner expertise, reinforcing a human-in-the-loop approach to athlete development and decision-making. Protecting sleep opportunities follows Cunha et al. (2023), while evaluating recovery through subsequent training responses addresses the distinction between data-collection feasibility and demonstrated benefit.

## Ethical/implementation issues

Responsible implementation requires understandable explanations of data collection and decisions, appropriate youth consent and assent, and defined access, retention, and withdrawal procedures. Athletes should be able to question interpretations. Accessible alternatives can address equipment, connectivity, and disability barriers. Staff must communicate uncertainty and avoid treating maturation estimates as definitive selection criteria. Acceptance, staff workload, and costs require evaluation alongside measurement accuracy.

## Future directions

Four priorities connect the collection. First, establish delayed retention and competitive transfer against credible conventional practice, documenting exposure and feedback schedules. Second, validate measurements across ages, sexes, maturation stages, para-sport, and resource settings. Leckey et al. (2025) highlight injury-prediction limitations; external validation and decision utility should precede deployment. Third, test recovery interventions during congested schedules, linking sleep and cognitive demands to performance and wellbeing. Fourth, evaluate implementation over full seasons, including autonomy and unintended effects. Future systems should also move beyond isolated metrics toward integrated models combining tactical, physical, physiological, psychological, and developmental information to support context-sensitive decision making. Transparent protocols and shareable code can support reproducibility while protecting personal data.
